# Interlamellar matrix governs human annulus fibrosus multiaxial behavior

**DOI:** 10.1038/s41598-020-74107-8

**Published:** 2020-11-09

**Authors:** Karim Kandil, Fahmi Zaïri, Tanguy Messager, Fahed Zaïri

**Affiliations:** 1 Unité de Mécanique de Lille (EA 7572 UML), Lille University, 59000 Lille, France; 2 Civil Engineering and geo-Environmental Laboratory (ULR 4515 LGCgE), Lille University, 59000 Lille, France; 3 Ramsay Générale de Santé, Hôpital privé Le Bois, 59000 Lille, France

**Keywords:** Health care, Medical research, Engineering, Materials science, Mathematics and computing

## Abstract

Establishing accurate structure–property relationships for intervertebral disc annulus fibrosus tissue is a fundamental task for a reliable computer simulation of the human spine but needs excessive theoretical-numerical-experimental works. The difficulty emanates from multiaxiality and anisotropy of the tissue response along with regional dependency of a complex hierarchic structure interacting with the surrounding environment. We present a new and simple hybrid microstructure-based experimental/modeling strategy allowing adaptation of animal disc model to human one. The trans-species strategy requires solely the basic knowledge of the uniaxial circumferential response of two different animal disc regions to predict the multiaxial response of any human disc region. This work demonstrates for the first time the determining role of the interlamellar matrix connecting the fibers-reinforced lamellae in the disc multiaxial response. Our approach shows encouraging multiaxial predictive capabilities making it a promising tool for human spine long-term prediction.

## Introduction

The intervertebral disc is the most critical body part due to its essential and mandatory role during the daily activities such as work, sport, walking or even while only standing up without any movement. It is always subjected to multiaxial loadings increasing its internal local stresses which could lead to disc damage, hernia and severe pain^1^. Because of the complex couplings existing between microstructure at different scales, intrinsic properties (viscosity and nonlinearity), internal fluid flow and external loading mode, the knowledge of stress distribution in disc needs to call computer simulations. Over the last four decades, it was an important subject of research but, to date, there is no disc computational model that introduces the entire set of features. The first contributions modeled the disc as an elastic tangled extracellular matrix (ECM) solid phase rigidified by elastic collagen fibers (CFs)^2–6^. Then, biphasic models appeared adding the fluid phase to the disc structure and taking into account its diffusion inside the disc^7–10^. Finally, biphasic models were extended in order to incorporate the ECM negative charges effect and their influence on the fluid movement and intradiscal osmotic pressure in the loaded and unloaded states^11–14^. In the most recent publications, the time-dependency of the soft tissue response is neglected. This is obviously not consistent with the viscoelastic insights of the different disc components^12,13,15^ but also with the fluid transfer inside and outside the disc that affects largely the biochemical volumetric behavior of the tissue^16–18^. As common weak points, most of recent models need plenty of experimental testing sets in order to calibrate, fit and design them. Moreover, as mentioned in the literature, it is not possible to predict the multiaxial behavior of the disc by means of uniaxial data^19,20^. As a consequence, for each local region of the disc a new experimental test should be established for the construction of each uniaxial and multiaxial model, and the identification of a new set of parameters corresponding to each test is required^19–22^ or a fitting procedure including all uniaxial and multiaxial data that leads to non-accurate results for both data^9,23,24^. Also, the construction of an accurate heterogeneous model taking into account the different radial and circumferential microstructure features of the disc requires exploring and identifying the corresponding related parameters of each region. No model until now is able to predict the uniaxial behavior of some disc regions based only on their microstructure components and to extend this behavior in order to predict the multiaxial response of the tissue. The actual models are able to estimate stress levels inside the disc with a completely wrong tissue volumetric deformation. In this regard, in the most recent models^17,18^, the interlamellar (ILM) matrix connecting the fibers-reinforced lamellae was introduced as a key structural factor of the inter-layer fluid transfer mechanism responsible for the tissue transversal behavior in the axial and radial disc directions. Strictly speaking, the structural contribution of this zone cannot be overlooked^25–32^ but its exact role in the disc biomechanics remains still mysterious.

This work addresses the problem of the multiaxial mechanics prediction of human disc. The main objective is to provide a quantitative prediction of the multiaxial response of a complete human disc with a minimum of experiments by means of a hybrid trans-species experimental/modeling strategy requiring the basic knowledge of the uniaxial circumferential response of only two different animal disc regions. Different model simulations are designed based upon well-known experimental tests issued from the literature in order to perfectly approve the model validity under identical conditions. As far as we know, a few experimental data are available in the literature for uniaxial stretching^33–35^, biaxial stretching^19,20^ and shearing^36^ tests often conducted under different testing conditions (Other publications could be found in the literature but they are limited to a single annulus region or present only one case of biaxial loading or shear response of a single shear mode). Our approach, fully three-dimensional, incorporates the different intrinsic and microstructure features that play a key role in the disc multiaxial response: ECM viscoelasticity, osmo-induced ECM swelling, CFs content/orientation and ILM matrix. Its principle advantage is its ability to be used almost under any experimental conditions reported in the open literature including variations in specimen shape and loading conditions in terms of strain rate and maximum strains in order to reproduce exactly the effectuated test. The model results are investigated and correlated to the different physiological movements and important conclusions are deduced.

## Results

### Uniaxial stretching path along with regional effects

**Figure 1:**
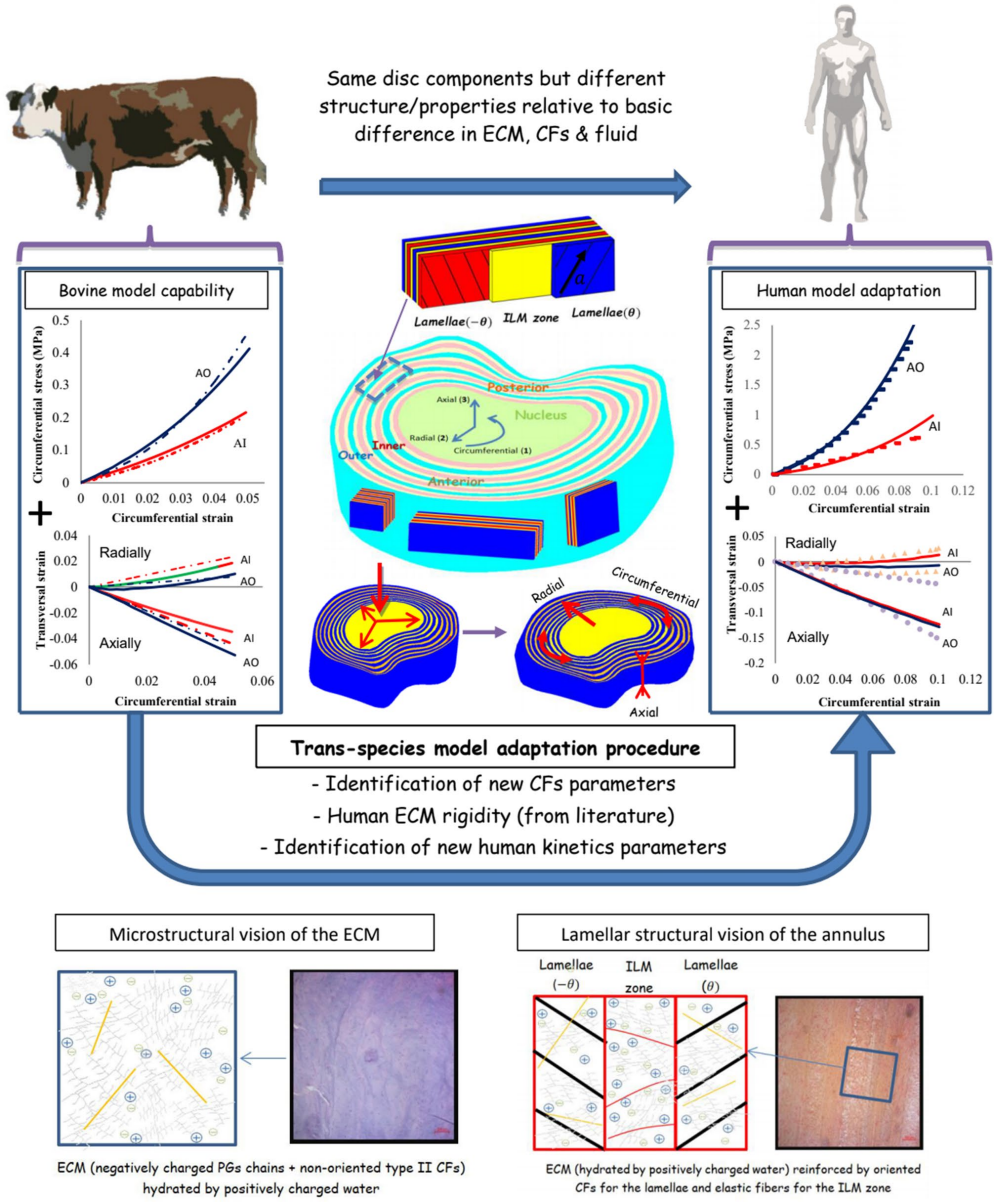
Hybrid experimental/modeling strategy for the trans-species transfer from bovine to human intervertebral disc model (solid lines: simulations, dashed lines/symbols: experiments). The microstructure-based model, identified using a very few data (only two uniaxial stress–strain responses in the circumferential direction at two disc regions and its related transversal strain history in axial and radial directions), provides the multiaxial quantitative predictions of all the human disc.

**Figure 2:**
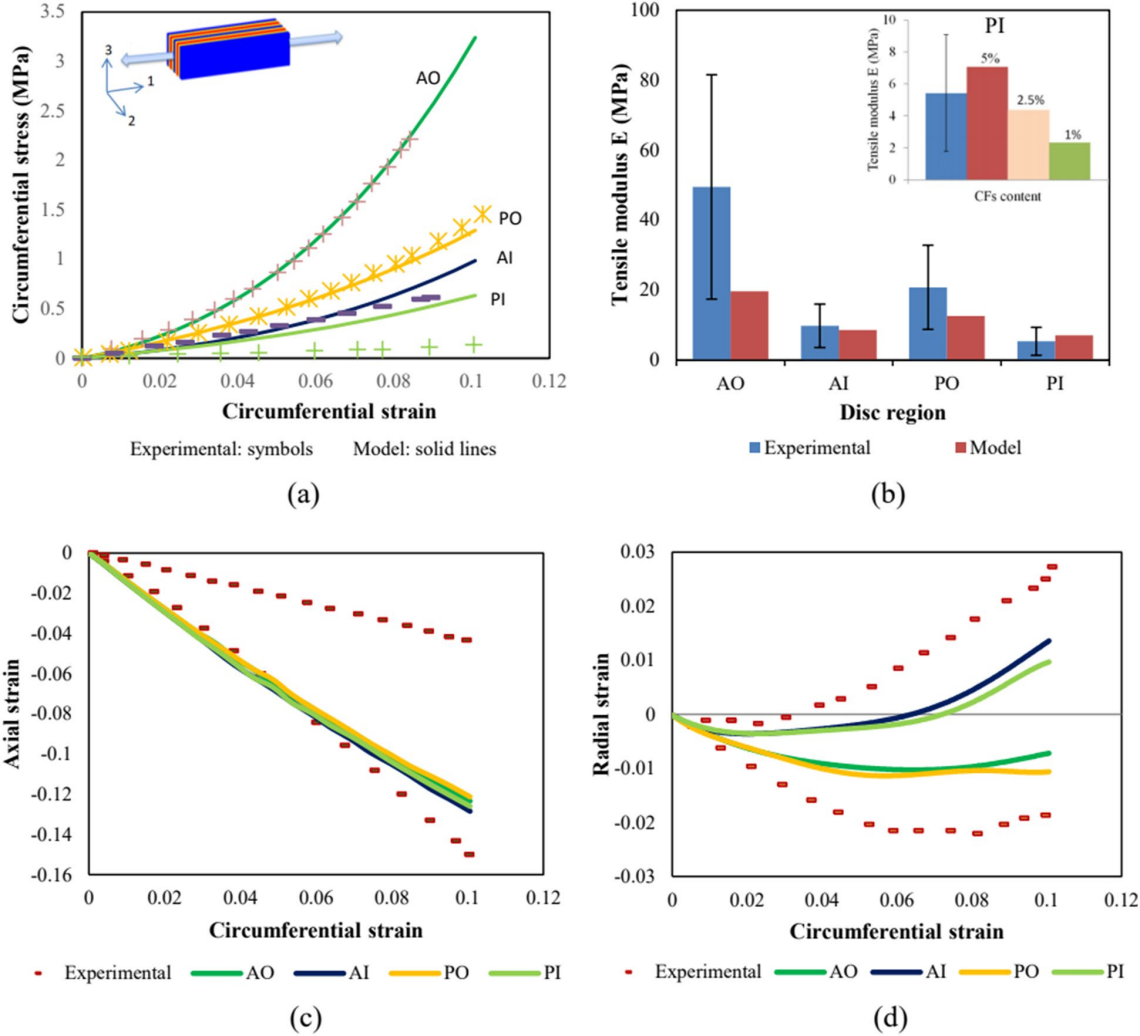
Uniaxial stretching model results in terms of (**a**) stress–strain responses and (**b**) tensile moduli compared to experimental data of Ebara et al.^34^, the insert shows PI predictions for different amounts of effective CFs, (**c**) transversal strain history in axial direction and (**d**) transversal strain history in radial direction compared to experimental data of Baldit et al.^16^. The figure shows the model fitting results for AO and AI to experimental data and the model predictions for PO and PI.

Uniaxial stretching responses were reproduced in-silico for specimens extracted from four different circumferential and radial disc regions (AO, PO, AI and PI) as shown in Fig. 1. The circumferential stress–strain and transversal responses of the four regions are reported in Fig. 2. The AO and AI regions were used to identify the fluid kinetics and the CFs parameters inside human intervertebral disc using a hybrid experimental/modeling strategy as described in Fig. 1. The latter will be discussed in details in “Methods” section. The response of the remaining disc regions, namely PO and PI, was then predicted by adapting well-known microstructure features in terms of CFs content/orientation and water content. The fitted and predicted numerical curves show an excellent agreement with the regional experimental results of Ebara et al.^34^ and Baldit et al.^16^. The behavior highlights a direct relation between the overall soft tissue mechanics, in terms of stiffness and Poisson’s ratio, and the microstructures along with the regional (circumferential and radial) variation in CFs content/orientation. The predicted slopes of the stress–strain curves of the posterior regions are lower than those obtained for the anterior regions. As well, the outer annulus is stiffer than the inner annulus. Quite interestingly, a very small radial deformation of the inner disc region compared to the outer disc region is obtained. The circumferential deformation is almost regional independent and its variation is negligible from a region to another. The very soft PI response reported for multilamellar or single lamellar specimens^34,35^ are impossible to catch with the model along CFs content is used as input data whereas it could almost be obtained by considering a non-fibrous ECM. The fibers content effect on the PI stiffness was investigated trying to find good explanations about this finding. An effective CFs content is suggested inspired from the microscopic observations of Tsuji et al.^37^ and Smith and Fazzalari^38^ who noticed a non-well-arranged CFs in the PI region compared to others. The stress–strain behavior was reproduced under different amounts of effective CFs and the corresponding stiffness was calculated and compared to the experimental results in Fig. 2. The effective CFs content shows a high ability to describe the low PI rigidity. Lower effective CFs content induces a lower rigidity modulus and a lower slope of the stress–strain conduct.

It is worth noticing that the model parameters were fitted using the uniaxial stretching data and the identified set of model parameters was used to generate the multiaxial predictions without any change or adjustment.

### Biaxial stretching paths

**Figure 3:**
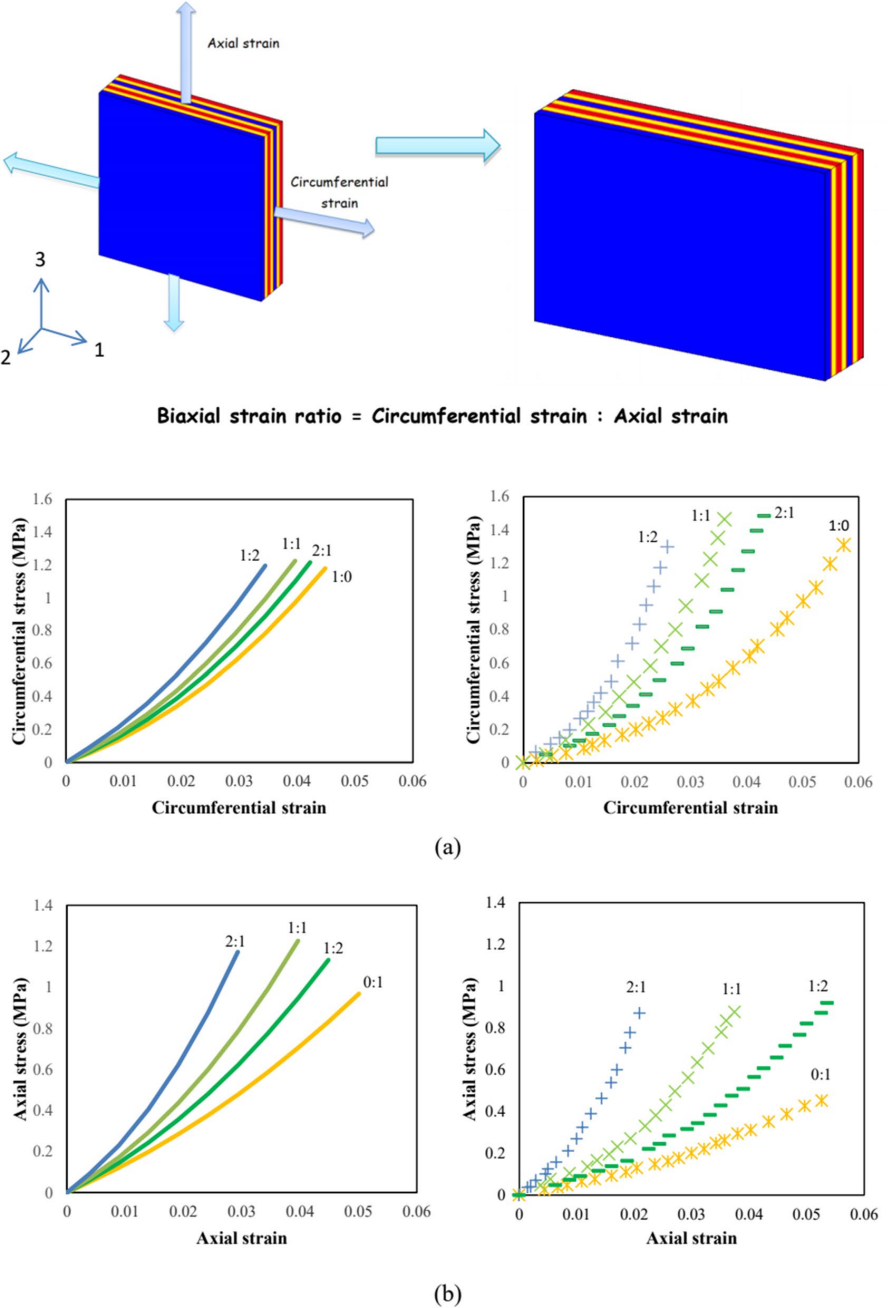
Biaxial stretching model results for different biaxial strain ratios (1:0, 0:1, 1:2, 2:1 and 1:1 where the two numbers refer to the circumferential and axial directions, respectively) in terms of (**a)** circumferential and (**b**) axial stress–strain responses compared to experimental data of O’Connell et al.^20^ (solid lines: simulations, symbols: experiments for AO specimens).

The predictions in terms of AO biaxial stretching responses are plotted for different biaxial strain ratios in Fig. 3. The in-silico results are compared to the typical in-vitro experimental results of O’Connell et al.^20^ reproducing the same experimental conditions. A global view at these plots shows that the rigidity of the specimen is affected by the biaxial strain ratio. The stress levels obtained under different biaxial cases are higher than the uniaxial stress levels. The strain ratio has a larger effect on the axial stresses than the circumferential ones. The experimental results show the same effect. For the strain ratios 1:0 and 0:1, the circumferential stress is higher than the axial stress. This is also observed for the strain ratio 1:2. For the strain ratio 2:1, a higher axial stress is obtained as also experimentally remarked.

### Shearing paths

**Figure 4:**
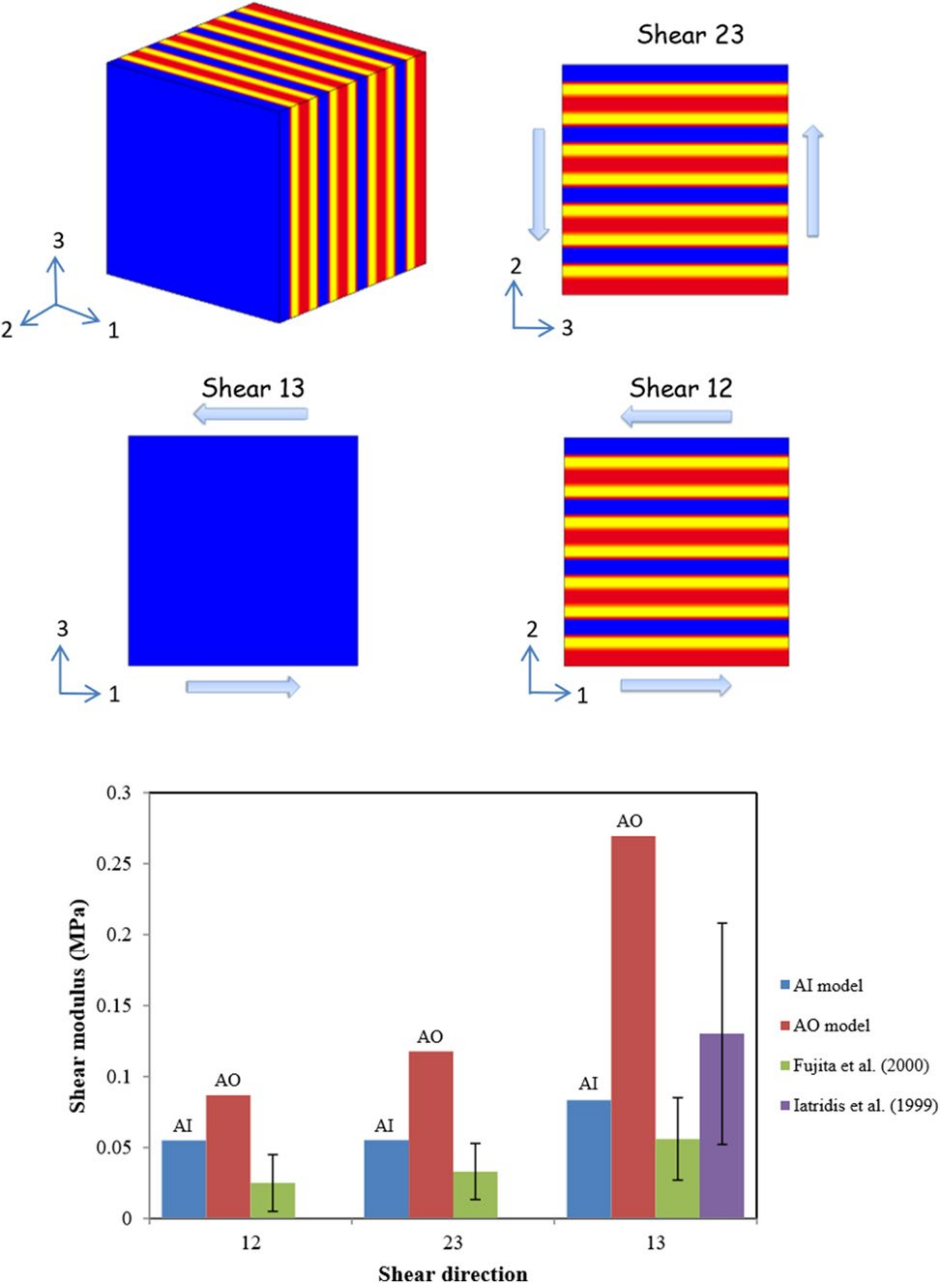
Shearing model results for different shear modes (12, 23 and 13) in terms of shear modulus compared to experimental data of Iatridis et al.^39^ and Fujita et al.^36^.

**Figure 5:**
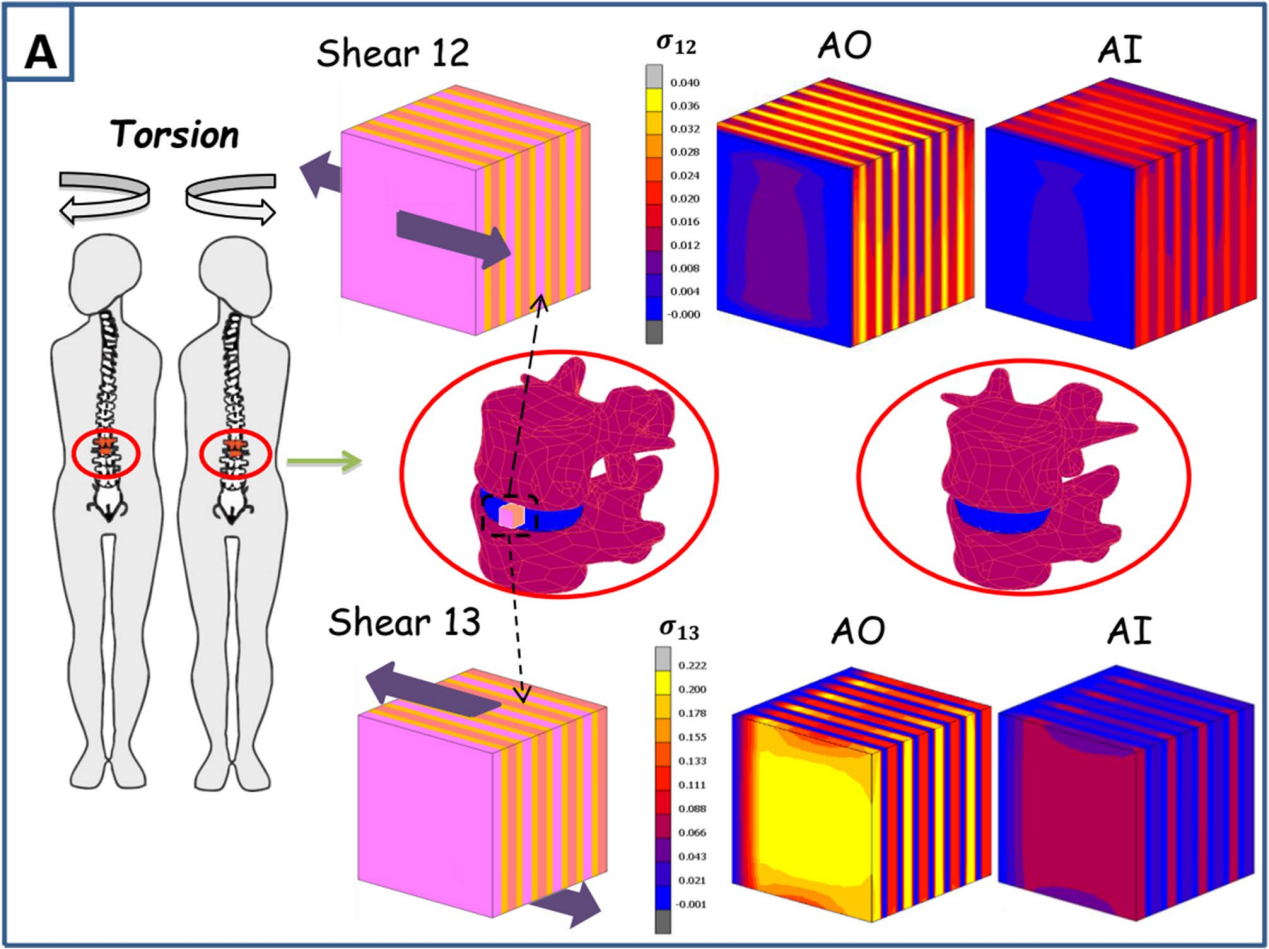

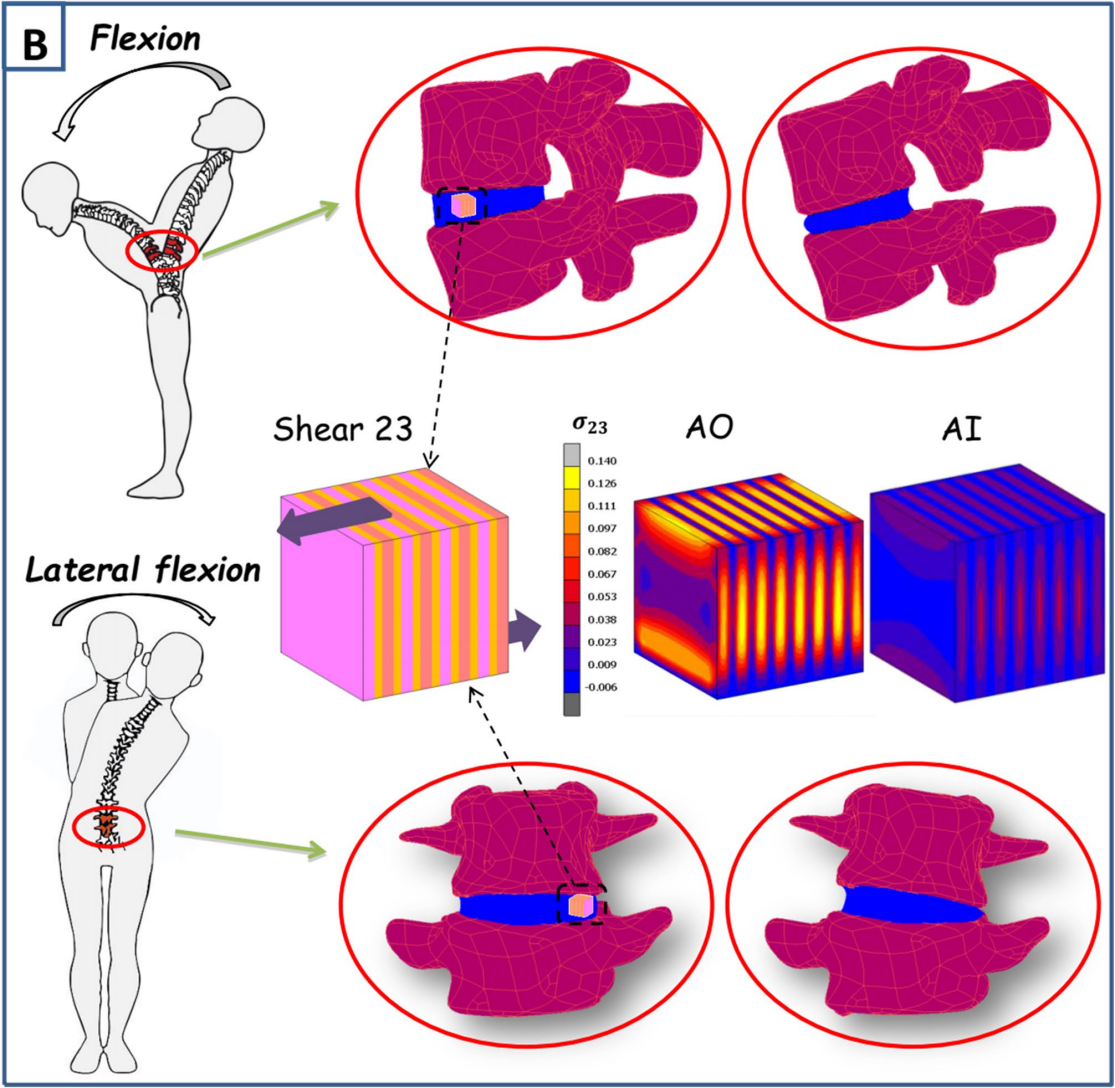
Correlation of the physiological movements with local shear stress fields in three-dimensional space of disc parts: (**A**) Shear modes 12 and 13 related to the torsional movement and (**B**) shear mode 23 related to the flexion and lateral flexion movements.

The annulus shearing responses were predicted for specimens subjected to the three different shear modes illustrated in Fig. 4. These loading modes translate the different loading cases occurring under torsional and bending physiological movements. The shear mode 12 corresponds to the interlamellar shear generated especially under spinal torsion due to the relative movements between lamellae and between the inner and outer annulus regions. The shear mode 13 corresponds to the shearing generated by the adjacent vertebrae on the annulus under torsional movement due to the relative motion between the upper and lower disc surfaces. The shear mode 23 corresponds to the shearing generated by the relative displacement of the inner and outer disc regions under especially flexion movement. The predicted shear modulus was computed under the different shear modes and compared to the experimental results of Iatridis et al.^39^ and Fujita et al.^36^. The in-silico results are found slightly higher than the experimental observations but the same trends are observed for the three modes. The mode 12 gives the lowest shear modulus and the mode 13 gives the highest shear modulus while the 23 shear modulus is in between. As well, the outer region shows higher shear modulus than the inner disc region. In order to analyze these modes locally, the local shear stress fields of the three modes are plotted in Fig. 5. Important insights are observed. For all the modes the local stress levels in the lamellae and ILM zones are higher in the outer disc region than the inner one. Under the 12 shear mode the highest stress levels are observed in the ILM zones unlike the 13 and 23 shear modes where the highest stresses are observed in the lamellae. While comparing the 23 and 13 modes we can notice that for the first one the highest stress concentrations are similar in all the lamellae but in the second one the highest concentrations are in the lamellae containing only fibers oriented towards the displacement direction. The contribution of the different local annulus zones to the shear disc mechanics under the daily physiological movements is investigated and the probability of damage initiation under these different modes is discussed.

## Discussion

The tensile stress–strain response of the annulus fibrosus is highly dependent on the regional variation of the CFs content/orientation^33,35^. Due to a higher CFs content, the outer annulus is stiffer than the inner annulus while the CFs angle difference leads to stiffer anterior regions than their posterior counterparts. This is a basic two-phase composite understanding in which the effective contributions of the ECM and the oriented CFs phases to the tensile stress–strain response are integrated. The regional variation of the negative charges content in the proteoglycans, decreasing from the nucleus to the outer disc region, leads to a variation in the ionic transfer by fluid exchange resulting in a variation of the transversal response translated by the Poisson’s ratio. The Poisson’s ratio related to the radial deformation is negative with higher values in the inner disc region compared to the outer disc region. Taking into account structural features and fluid exchange allows simulating the auxetic response of the tissue. In the meantime, the Poisson's ratio related to the axial deformation is not affected by the microstructure features and is almost the same for all the studied disc regions. The model shows that the Poisson’s ratio is principally influenced by the water content and the swelling of the ILM zones. As well, the presence of the high CFs content in the outer disc region compared to the inner one retards more the fluid flow and decreases its content which affects in turn the tissue swelling and the radial strains. On the other side, the Poisson's ratio related to the axial deformation is higher than 1 for the four regions and it is less sensible to the microstructure variation compared to the Poisson’s ratio related to the radial deformation, its value being relatively constant throughout the annulus. The PI region has experimentally the lowest rigidity that could correspond to the non-fibrillar ECM modulus due to the non-well-arranged and high dispersion of CFs^37,38^. The calculation of an effective oriented fibers content relating not only the fibers volume fraction but also considering their arrangement helped to vary the rigidity and to reach the low rigidity levels.

Our hybrid experimental/modeling strategy leads to drastically reduce the amount of experimental work needed to characterize the complete human disc behavior thanks to an accurate and effective chemo-mechanical microstructure-based approach. Modeling the annulus uniaxial stretching in the circumferential direction (representative of the disc compression mechanics) gives a lot of fruitful information and new explanations about the disc chemo-mechanical interactions as well as the different phenomena occurring in the disc. Nonetheless, predicting more realistic and complex spine movements requires the simulation of accurate annulus behavior under multiaxial (biaxial and shear) loadings.

Because it is constrained radially by the nucleus and axially by the adjacent vertebrae, the biaxial deformation of the annulus represents a more realistic loading case. The circumferential and axial stresses under different biaxial strain levels and strain ratios give similar results than those previously experimentally observed in the literature^20^. The annulus rigidity under different biaxial cases are higher than the uniaxial rigidity and the stress levels under the same maximum strain are relatively higher which have been also noticed experimentally^19,40^. The strain ratio shows as well a high effect on the biaxial behavior of the annulus especially on the axial stress. Many recent numerical and analytical models tried to compute the shear stress inside the disc under uniaxial loadings^29,41^ due to their simplicity. Only one recent contribution^14^ succeeded to reproduce the multiaxial behavior of the annulus based on the uniaxial behavior of the disc. Unfortunately, this contribution considered only the preloading free swelling effect and did not take into account the chemical coupling inside the disc with the surrounding environment and the related time-dependent behavior under mechanical loading that is always present during the physiological complex movements^18^. This coupling affects largely the transversal deformations which could lead to completely wrong results especially under biaxial tests. As well, it is considered that the Poisson’s ratios of the annulus related to axial and radial directions are constant and their values are between 0 and 0.5 which is proven experimentally to be wrong^16–18^. The annulus behavior under shearing is very important and could be of a prime interest while studying the disc biomechanics under especially bending and torsion movements. The latter are common mechanical loadings during the different daily spinal motions^42^ and generate shearing inside the disc that could lead to many disc dysfunctions when combined with other axial loads. Numerically, the 12 shear modulus is lower than the 23 shear modulus which is also lower than the 13 shear modulus. All moduli of the outer specimens are found higher than the internal ones for the same shear mode. The latter difference is confirmed by the same experimental conduct observed in the literature^36,43^. The shear predictions reveal very interesting observations. The lowest shear stress levels are observed under the 12 shear mode but occurred throughout the disc with a higher concentration in the non-fibrillar ILM zones that are much weaker than the adjacent lamellae increasing the risk of annulus delamination. This has been also observed experimentally in many contributions in the literature suggesting the contribution of especially the ILM connections and the lamellar fiber matrix interactions to this shearing mode^43^. The highest stresses for the 23 and 13 shear modes are observed in the lamellae. Nonetheless, only the lamellae with fibers oriented towards the loading direction are subjected to the maximum stress values in the mode 13. Under the 23 shear mode all the lamellae are subjected to those high stresses with higher maximum stress values compared to those observed in the 13 shear mode. This could be explained by the relation between the shear modulus of these two modes and the oriented CFs that was observed in previous experimental contributions^43^. While relating these shear modes to physiological movements we could notice that the 12 and 23 shear modes are more related to the torsional movements produced under twisting body actions as shown in Fig. 5 generating the highest stresses in the lamellae and the ILM zones as well. The 23 shear mode, related to bending and lateral bending body movements, generates moderate stress levels in the lamellae and very low stresses in the ILM zones. These results highlight for the same shear strains the high damage risk under torsional body movements making it the most dangerous and risky multiaxial move of the body. This is not in accordance with the experimental observations of Costi et al.^44^ who investigated the maximum shear strain suggesting that the lateral bending and flexion are the most dangerous movements affecting the disc due to their high physiological values compared to axial torsion for the lumbar spine. These contradictory results reveal the importance of making more complex calculations combining the two studies and measuring the stress levels under the maximum physiological shear strains and taking as well into consideration the local maximum shear stress levels. Indeed, lower maximum shear stress levels could be observed globally in the specimen under a certain mode with high stresses affecting the weak ILM zones that exhibit a lower ultimate stress due to the absence of fibers.

We can conclude that our modeling approach succeeds to predict the multiaxial behavior of the annulus by means of uniaxial experimental data which was supposed to be not possible in the previous literature^19,20^. The use of a layered model with distinct lamellar and interlamellar layers is mandatory to access the core of the annulus fibrosus and to define their respective role in the disc mechanics. By such a modeling approach, we can identify the critical zones where a likely higher risk of local damage is present under complex external mechanical loadings. All previous layered models considered these zones as common sliding zones separating the lamellae but the role of the ILM zones does not stop trivially there. Their essential role as chemical actuator of the transfer fluid through the annulus tissue and the corresponding unusual transversal behavior was only appreciated through very recent contributions^17,18,45^. In the present paper, we highlighted for the first time the determining role of the ILM zones on the multiaxial response of the annulus.

As a final point of discussion, let us come back to the mechanics of the whole disc. The compression of the whole disc (due in-vivo to muscular tension and body weight) provokes the uniaxial circumferential stretching of the annulus due to swelling of the nucleus (inner gel-like disc portion), combined to multiaxial mechanical paths during body movements. Although in-vitro experiments allowed to bring insights on the mechanics of the whole disc^46–48^, the annulus/nucleus interaction under complex external mechanical loadings is still an open issue. The construction of a whole disc model including our annulus model (with the regional effects) and the explicit presence of the nucleus would be useful for a thorough understanding of the functionality of the disc and its local behavior. The annulus structure-response relationship varies between individuals, between the different discs of the same spinal column and depends on the degeneration state of the disc. Therefore, it would be useful in the continuity of the model development to generate patient-specific models based on a hybrid methodology combining our modeling approach and direct MRI measurements on functional spine units.

## Methods

### Computational models

Uniaxial stretching, biaxial stretching and shearing are all considered in this work. The three sets of experiments are replicated in-silico using the finite element method according to the experimental recommendations available in the literature regarding specimen shapes, sizes and mechanical conditions in terms of strain rate and maximum strain. As observed in the works of Gregory and Callaghan^49^ and Derrouiche et al.^45^ the strain rate has an effect on the stress–strain and transversal responses of the multilamellar annulus specimen. As a consequence, for all the simulated tests, quasi-static conditions were ensured by using strain rates of 0.002 s^−1^ and 0.0002 s^−1^ depending on the strain rate level of the reproduced experimental test. The maximum strain used in all the simulations varies between 5 and 10% which corresponds to the maximum physiological strain level observed experimentally for annulus under compression^44,50^. The latter values have been often used in many experimental studies^16,34^. All the geometrical models were meshed using isoparametric and arbitrarily hexahedrics 8-node meshing elements. An adequate element size is chosen to ensure mesh-independency. All the ILM-lamellar interfaces of the stratified soft tissue are considered perfect. The structural features used as model inputs for the different human disc regions are reported in Table 1 and are extracted from well-documented papers of the literature^35,51–54^.

**Table 1:** Structure features of the human disc.

	AO	PO	AI	PI
Fibers content	${12}.{5}\%$	${12}.{5}\%$	$5\%$	$5\%$
Fibers orientation	${21}^\circ$	$45^\circ$	$35^\circ$	${45}^\circ$
Water content	$74\%$	$74\%$	$80\%$	$80\%$

The fibers content decreases radially while getting closer to the nucleus^51–53^. The fibers orientation increases and tends towards the spinal axial direction while getting closer to the nucleus^35^. The water content increases radially in the disc while getting closer to the nucleus. A non-significant water content difference exists between the posterior and anterior regions^54^.

### Uniaxial stretching conditions and designed material kinetics

A computational model was designed as described by Kandil et al.^18^ for the uniaxial conditions. Due to the large specimen size and large calculation time, a representative elementary volume (REV) of the specimen was designed in order to reproduce the in-vitro experiments of Ebara et al.^34^ and Baldit et al.^16^. A REV of 2 × 2 × 1.6 mm is modeled. The same REV was used to model the experiments of the two papers. Similar boundary conditions were applied but different symmetry conditions were considered in each case. For the Baldit et al.^16^ specimen, the corresponding modeled REV is one-fourth of the specimen cross-section and one-sixth of the specimen height and for the Ebara et al.^34^ specimen, it corresponds to one-half of the specimen cross-section and one-sixth of the specimen height.

Experimentally-based kinetics has been designed by Kandil et al.^18^ using the bovine model. The human disc contains a lower CFs content and a slightly higher water content compared to bovine disc^51,55^. As illustrated in Fig. 1, a hybrid experimental/modeling strategy for the trans-species transfer from bovine to human intervertebral disc model is adopted via a three-step methodology to reach the final result of model vs. experiments and the related predictions. In the first step, the fluid transfer equations, first developed for bovine annulus by Kandil et al.^18^, were adapted to the human annulus by adjusting the fluid kinetics on the human (AO and AI) experimental transversal curves of Baldit et al.^16^ shown in Fig. 1. In the second step, the fiber intrinsic mechanical parameters were identified using the human (AO and AI) experimental stress–strain curves of Ebara et al.^34^. In the third step, the predictions were made possible in the remaining posterior (PO and PI) regions by applying the related fibers content/orientation and water concentration.

### Biaxial stretching conditions

Different biaxial loading cases were simulated with the same specimen dimensions reported for the in-vitro experiments of O’Connell et al.^20^: 7 × 7 × 1.6 mm. The computational models were subjected to different strain loading ratios (= circumferential strain : axial strain, {1:0, 0:1, 1:2, 2:1 and 1:1}) along with a maximum strain of 5% and a strain rate of 0.0002 s^−1^. Two sets of tests were established. The first one consisted on circumferential stress calculation while the second one on axial stress calculation, both under the different loading ratios.

### Shearing conditions

Three shear modes were simulated following the boundary conditions of Fujita et al.^36^: 12, 13 and 23 as shown in Fig. 4 using 3.33 mm cubes subjected to a maximum shear strain of 5% and a shear strain rate of 0.0002 s^−1^. Because the in-vitro experiments of Fujita et al.^36^ were performed on anterior specimens but without mentioning their exact radial positions, both AO and AI computational models were considered. Due to a negligible fluid transfer upon shearing, the latter was not considered in the computational models.

### Microstructure-based chemo-mechanical constitutive model

In what follows, the key points of the chemo-mechanical model are briefly summarized. The model is fully three-dimensional and considers the time-dependency of the annulus fibrosus tissue response in relation to the microstructure, the intrinsic features and the chemical-induced internal fluid variation.

#### Kinematics

Starting point for the derivation of any finite-strain constitutive model is the kinematics within the framework of nonlinear continuum mechanics. If ${\varvec{x}}$ is the current position of a material point of the continuum body located at ${\varvec{X}}$ in the initial configuration, the deformation gradient tensor is: ${\mathbf{F}} = {{\partial {\varvec{x}}} \mathord{\left/ {\vphantom {{\partial {\varvec{x}}} \partial }} \right. \kern-\nulldelimiterspace} \partial }{\varvec{X}}$. The time derivative is defined as: ${\dot{\mathbf{F}}} = {\mathbf{LF}}$ in which ${\mathbf{L}} = {{\partial {\varvec{v}}} \mathord{\left/ {\vphantom {{\partial {\varvec{v}}} {\partial {\varvec{x}}}}} \right. \kern-\nulldelimiterspace} {\partial {\varvec{x}}}}$ is the spatial velocity gradient tensor with ${\varvec{v}} = {{\partial {\varvec{x}}} \mathord{\left/ {\vphantom {{\partial {\varvec{x}}} {\partial t}}} \right. \kern-\nulldelimiterspace} {\partial t}}$. Let us consider an intermediate virtual configuration for the chemical coupling with the surrounding environment. This concept allows to further multiplicatively split the tensor ${\mathbf{F}}$ into two contributions: ${\mathbf{F}} = {\mathbf{F}}_{mech} {\mathbf{F}}_{chem}$ in which ${\mathbf{F}}_{mech}$ is the mechanical part and ${\mathbf{F}}_{chem}$ is the chemical-induced volumetric part. Using a similar sequence of configurations with an intermediate virtual configuration during a spontaneous elastic unloading, the mechanical deformation gradient tensor ${\mathbf{F}}_{mech}$ is in turn multiplicatively split into an elastic part ${\mathbf{F}}_{e}$ and a viscous part ${\mathbf{F}}_{v}$ such as: ${\mathbf{F}}_{mech} = {\mathbf{F}}_{e} {\mathbf{F}}_{v}$ in the aim representing the intrinsic viscosity of the ECM including all non-fibrillar “solid” components. In view of the mechanical incompressibility of all “solid” components (ECM and CFs), the determinants (Jacobian) of the viscous and elastic deformation gradients are: $J_{{\text{v}}} = \det \left( {{\mathbf{F}}_{{\text{v}}} } \right) = 1$ and $J_{{\text{e}}} = \det \left( {{\mathbf{F}}_{{\text{e}}} } \right) = 1$. The chemical-induced volumetric part is: ${\mathbf{F}}_{chem} = {\mathbf{I}}J^{{{1 \mathord{\left/ {\vphantom {1 3}} \right. \kern-\nulldelimiterspace} 3}}}$ in which ${\mathbf{I}}$ is the unit tensor and $J = \det \left( {\mathbf{F}} \right) > 0$ is the determinant of the deformation gradient tensor, i.e. the tissue volumetric deformation. The spatial velocity gradient tensor ${\mathbf{L}}$ is additively spit into a mechanical part ${\mathbf{L}}_{mech}$ and a chemical part ${\mathbf{L}}_{chem}$ as: ${\mathbf{L}} = {\mathbf{L}}_{mech} + {\mathbf{L}}_{chem}$ with ${\mathbf{L}}_{chem} = {\mathbf{F}}_{mech} {\dot{\mathbf{F}}}_{chem} {\mathbf{F}}_{chem}^{ - 1} {\mathbf{F}}_{mech}^{ - 1} = {\mathbf{I}}{{\dot{J}} \mathord{\left/ {\vphantom {{\dot{J}} {3J}}} \right. \kern-\nulldelimiterspace} {3J}}$ and ${\mathbf{L}}_{mech} = {\dot{\mathbf{F}}}_{mech} {\mathbf{F}}_{mech}^{ - 1} = {\mathbf{L}}_{e} + {\mathbf{L}}_{v}$ in which ${\mathbf{L}}_{e} = {\dot{\mathbf{F}}}_{e} {\mathbf{F}}_{e}^{ - 1}$ is the elastic part and ${\mathbf{L}}_{v} = {\mathbf{F}}_{e} {\dot{\mathbf{F}}}_{v} {\mathbf{F}}_{v}^{ - 1} {\mathbf{F}}_{e}^{ - 1}$ is the viscous part that in turn may be written as: ${\mathbf{L}}_{v} = {\mathbf{D}}_{v} + {\mathbf{W}}_{v}$ in which ${\mathbf{D}}_{v} = {{\left( {{\mathbf{L}}_{v} + {\mathbf{L}}_{v}^{T} } \right)} \mathord{\left/ {\vphantom {{\left( {{\mathbf{L}}_{v} + {\mathbf{L}}_{v}^{T} } \right)} 2}} \right. \kern-\nulldelimiterspace} 2}$ is the (symmetric) viscous stretching rate tensor and ${\mathbf{W}}_{v} = {{\left( {{\mathbf{L}}_{v} - {\mathbf{L}}_{v}^{T} } \right)} \mathord{\left/ {\vphantom {{\left( {{\mathbf{L}}_{v} - {\mathbf{L}}_{v}^{T} } \right)} 2}} \right. \kern-\nulldelimiterspace} 2}$ is the (skew-symmetric) viscous spin rate tensor. The common assumption of viscous irrotationality is applied with no loss in generality^56^, i.e. ${\mathbf{W}}_{v} = {\mathbf{0}}$ and ${\mathbf{D}}_{v} {\mathbf{ = }}{\mathbf{L}}_{v}$. The different contributions of the right and left Cauchy-Green deformation tensors, ${\mathbf{C}} = {\mathbf{F}}^{{T}} {\mathbf{F}}$ and ${\mathbf{B}} = {\mathbf{FF}}^{{T}}$, are: ${\mathbf{C}}_{mech} = {\mathbf{F}}_{mech}^{T} {\mathbf{F}}_{mech}$, ${\mathbf{B}}_{mech} = {\mathbf{F}}_{mech} {\mathbf{F}}_{mech}^{T}$, ${\mathbf{C}}_{e} = {\mathbf{F}}_{e}^{T} {\mathbf{F}}_{e}$, ${\mathbf{B}}_{e} = {\mathbf{F}}_{e} {\mathbf{F}}_{e}^{T}$, ${\mathbf{C}}_{v} = {\mathbf{F}}_{v}^{T} {\mathbf{F}}_{v}$, ${\mathbf{B}}_{v} = {\mathbf{F}}_{v} {\mathbf{F}}_{v}^{T}$, ${\mathbf{C}}_{chem} = J^{2/3} {\mathbf{I}}$ and ${\mathbf{B}}_{chem} = J^{2/3} {\mathbf{I}}$.

#### Constitutive equations

In the aim of attributing a constitutive relationship to each main component (ECM, CFs and fluid) of the annulus fibrosus tissue, the free energy function $\psi$ is additively split via the volume fraction concept:

1 $$ \psi = \left( {1 - \phi_{CF} } \right)\psi_{ECM} + \phi_{CF} \psi_{CF} + \left( {1 - \phi_{CF} } \right)\psi_{chem} $$

where $\phi_{CF}$ is the CFs content, $\psi_{ECM}$ is the ECM free energy function, $\psi_{CF}$ is the CFs free energy function and $\psi_{chem}$ is the chemical-induced volumetric free energy function, respectively, given by:

2 $$ \psi_{ECM} = - \frac{E}{6}I_{1}^{\max } \ln \left( {1 - \frac{{I_{1} - 3}}{{I_{1}^{\max } }}} \right) - \frac{{E_{v} }}{6}I_{1v}^{\max } \ln \left( {1 - \frac{{I_{1e} - 3}}{{I_{1v}^{\max } }}} \right) $$

3 $$ \psi_{CF} = A_{1} \left( {I_{4} - 1} \right) + A_{2} \left( {I_{4} - 1} \right)^{2} - 2A_{1} \ln \left( {\lambda_{I}^{{x^{2} }} \lambda_{II}^{{y^{2} }} \lambda_{III}^{{z^{2} }} } \right) $$

4 $$ \psi_{chem} = \frac{1}{4}k\left( {J^{2} - 1 - 2\ln J} \right) $$

The free energy function (2) corresponds to a Gent formulation^57^ in which an additive split into elastic and inelastic contributions is considered. The terms $I_{1} = {\text{tr}}{\mathbf{B}}_{mech}$ and $I_{1e} = {\text{tr}}{\mathbf{B}}_{e}$ are the first invariants. The free energy function (3) introduces $I_{4} = {\varvec{a}}{\mathbf{C}}_{mech} {\varvec{a}}$ as the fourth invariant where ${\varvec{a}} = x{\varvec{e}}_{1} + y{\varvec{e}}_{2} + z{\varvec{e}}_{3}$ is the unit vector in the initial configuration^58^ (see Fig. 1). The terms $\lambda_{I} = \sqrt {{\varvec{e}}_{1} {\mathbf{C}}_{mech} {\varvec{e}}_{1} }$, $\lambda_{II} = \sqrt {{\varvec{e}}_{2} {\mathbf{C}}_{mech} {\varvec{e}}_{2} }$ and $\lambda_{III} = \sqrt {{\varvec{e}}_{3} {\mathbf{C}}_{mech} {\varvec{e}}_{3} }$ are the stretches along the CFs principal axes. Remind that ILM is a non-fibrillar zone, i.e. $\phi_{CF}$ = 0 in ILM. The material constants are the ECM tensile modulus $E$, the ECM limiting extensibility constant $I_{1}^{\max }$, the ECM viscous constants $E_{v}$ and $I_{1v}^{\max }$, the CFs constants $A_{1}$ and $A_{2}$, and the bulk modulus $k$.

The set of free energy functions (2)–(4) forms the basis of a suitable theory to constitutively coordinate the dual stress and strain tensors:

5 $$ {{\varvec{\upsigma}}} = \left( {1 - \phi_{CF} } \right){{\varvec{\upsigma}}}_{ECM} + \phi_{CF} {{\varvec{\upsigma}}}_{CF} + \left( {1 - \phi_{CF} } \right){{\varvec{\upsigma}}}_{chem} $$

where the mechanical-based Cauchy stresses in the ECM and CFs, ${{\varvec{\upsigma}}}_{ECM}$ and ${{\varvec{\upsigma}}}_{CF}$, are deduced from the differentiation of the free energy functions (2) and (3) with respect to the corresponding deformations:

6 $$ {{\varvec{\upsigma}}}_{ECM} = {\frac {2}{J}}{\mathbf{F}}_{mech} \frac{{\partial \psi_{ECM} }}{{\partial {\mathbf{C}}_{mech} }}{\mathbf{F}}_{mech}^{T} + {\frac {2}{J}}{\mathbf{F}}_{e} \frac{{\partial \psi_{ECM} }}{{\partial {\mathbf{C}}_{e} }}{\mathbf{F}}_{e}^{T} \;{\text{and}}\;{{\varvec{\upsigma}}}_{CF} = {\frac {2}{J}}{\mathbf{F}}_{mech} \frac{{\partial \psi_{CF} }}{{\partial {\mathbf{C}}_{mech} }}{\mathbf{F}}_{mech}^{T} $$

in which the first term of the left formula corresponds to the elastic Cauchy stress ${{\varvec{\upsigma}}}_{e}$ and the second term corresponds to the viscous Cauchy stress ${{\varvec{\upsigma}}}_{v}$, such that ${{\varvec{\upsigma}}}_{ECM} = {{\varvec{\upsigma}}}_{e} + {{\varvec{\upsigma}}}_{v}$. Note that the mechanical incompressibility of all “solid” components (ECM and CFs) means that the mechanical-based Cauchy stresses in the ECM and CFs are traceless tensors, i.e. ${\text{tr}}\left( {{{\varvec{\upsigma}}}_{e} } \right) = 0$, ${\text{tr}}\left( {{{\varvec{\upsigma}}}_{v} } \right) = 0$ and ${\text{tr}}\left( {{{\varvec{\upsigma}}}_{CF} } \right) = 0$. The chemical-based Cauchy stress ${{\varvec{\upsigma}}}_{chem} = p{\mathbf{I}}$ ($p = {{{\text{tr}}\left( {{\varvec{\upsigma}}} \right)} \mathord{\left/ {\vphantom {{{\text{tr}}\left( {{\varvec{\upsigma}}} \right)} 3}} \right. \kern-\nulldelimiterspace} 3}$ being the hydrostatic pressure) is deduced from the differentiation of the free energy function (4) with respect to the tissue volumetric deformation:

7 $$ {{\varvec{\upsigma}}}_{chem} = \frac{{\partial \psi_{chem} }}{\partial J}{\mathbf{\text {I}}} $$

The driving force for the viscous Cauchy stress ${{\varvec{\upsigma}}}_{v}$ is the elastic deformation gradient tensor ${\mathbf{F}}_{e} = {\mathbf{F}}_{mech} {\mathbf{F}}_{v}^{ - 1}$, the viscous deformation gradient tensor ${\mathbf{F}}_{v}$ being computed via ${\dot{\mathbf{F}}}_{v} {\mathbf{ = F}}_{e}^{ - 1} {\mathbf{D}}_{v} {\mathbf{F}}_{mech}$. The viscous stretching rate tensor ${\mathbf{D}}_{v}$ is described by the following flow rule: ${\mathbf{D}}_{v} = \dot{\gamma }_{v} {{{\varvec \sigma^{\prime}}_{v} } \mathord{\left/ {\vphantom {{{\sigma^{\prime}}_{v} } {\sqrt 2 \left\| {{{\varvec{\upsigma}}}_{v} } \right\|}}} \right. \kern-\nulldelimiterspace} {\sqrt 2 \left\| {{{\varvec{\upsigma}}}_{v} } \right\|}}$ where $\left\| {{{\varvec{\upsigma}}}_{v} } \right\| = \sqrt {{{{\text{tr}}\left( {{\varvec \sigma^{\prime}}_{v} {\varvec \sigma^{\prime}}_{v} } \right)} \mathord{\left/ {\vphantom {{{\text{tr}}\left( {{\mathbf{\sigma^{\prime}}}_{v} {\mathbf{\sigma^{\prime}}}_{v} } \right)} 2}} \right. \kern-\nulldelimiterspace} 2}}$ is the effective value of the viscous Cauchy stress ${{\varvec{\upsigma}}}_{v}$, ${\varvec \sigma^{\prime}}_{v}$ is the deviatoric part of ${{\varvec{\upsigma}}}_{v}$ and $\dot{\gamma }_{v}$ is the accumulated viscous strain rate. Invoking a viscoplasticity with no threshold^59^, the following expression is retained^60^:

8 $$ \dot{\gamma }_{v} = d\left| {\sqrt {{{I_{1v} } \mathord{\left/ {\vphantom {{I_{1v} } 3}} \right. \kern-\nulldelimiterspace} 3}} - 1} \right|^{ - m} \left\| {{{\varvec{\upsigma}}}_{v} } \right\| $$

where $d$ is the ECM viscous multiplier constant and $m$ is the ECM viscous stretch-dependency constant. The term $I_{1v} = {\text{tr}}{\mathbf{B}}_{v}$ is the first invariant of the left Cauchy-Green strain tensor ${\mathbf{B}}_{v} = {\mathbf{F}}_{v} {\mathbf{F}}_{v}^{T}$.

The fluid flow governs the transversal strains (radially and axially) and is responsible of the unusual Poisson's ratios of the tissue. For this reason accurate experimentally-based fluid kinetics is required, which is the missing point of all available papers in the literature. The adopted fluid flow kinetics is defined by the Jacobian $J$ as follows:

9 $$ J = n_{{f_{m} }} \xi \,\eta $$

in which $\Delta J$ represents the tissue swelling function controling the fluid movement inside the disc under mechanical loading, $n_{{f_{m} }}$ is the internal fluid content, $\xi$ is a dimensionless transportation coefficient, $\eta$ is a dimensionless free swelling coefficient (equal to 0.5 in the case of physiological salt condition) and $n_{{f_{m} }}$ is the internal fluid content. The latter is controlled by:

10 $$ \dot{n}_{{f_{m} }} = \beta_{m} \left( {1 - \frac{{n_{{f_{m} }} }}{{n_{{f_{lim} }} }}} \right) $$

where $\beta_{m}$ is the fluid flow constant and $n_{{f_{lim} }}$ is the maximum fluid content that could be reached inside the disc.

The constitutive model has been implemented into the finite element code MSC.Marc by means of a set of subroutines. The main calculation steps of the model implementation are provided elsewhere^18^. The reader is also referred to complementary references^61,62^ for the general implementation procedure of coupled models.

#### Parameters identification

The model parameters and the material kinetics have been determined using experimental data in order to represent the human disc annulus response in connection to microstructure differences in the different disc regions (Table 1). The experimentally-based fluid flow kinetics is guided by the applied mechanical loading and the osmotic effect. Also, due to the fibers presence retarding the fluid movement in lamellae and the difference of the fixed charges density in the two zones, ILM and lamellae behave differently. The values of $\beta_{m}$ are, respectively, for the lamellae and ILM zones:

11 $$ \beta_{{m_{lam} }}^{{_{{}} }} = - \;0.000{\text{4 s}}^{{ - {1}}} \;{\text{and}}\;\beta_{{m_{ILM} }} = 0.000{\text{97 s}}^{{ - {1}}} $$

The transportation coefficient $\xi$ is a function of the strain rate $\dot{\varepsilon }$ and is described by the following equations for the lamellae and ILM zones:

12 $$ \xi_{{AI_{lam} }} = \xi_{{PI_{lam} }} = {2}0{328}{\dot{\varepsilon}} - 0.{4}0{56}\;{\text{and}}\;\xi_{{AO_{lam} }} = \xi_{{PO_{lam} }} {13283}\dot{\varepsilon } - 0.{1767} $$

13 $$ \xi_{{AI_{ILM} }} = \xi_{{PI_{ILM} }} = {7455}.{6}{\dot{\varepsilon}} + 0.{3789}\;{\text{and}}\;\xi_{{AO_{ILM} }} = \xi_{{PO_{ILM} }} = {3719}.{4}\dot{\varepsilon } + 0.{2}0{11} $$

The difference in the inner and outer disc volumetric responses is translated by different bulk moduli:

14 $$ k_{AI} = k_{PI} = 800\;{\text{MPa and }}k_{AO} = k_{PO} = 3500\;{\text{MPa}} $$

Note that the fluid kinetics of Kandil et al.^18^ is found able to reproduce the typical transversal strain history reported by Baldit et al.^16^. Similar trends are obtained with the newly identified human parameters due to the close water content between human and bovine. Although the ECM stiffness could vary from a disc region to another, it was chosen to be the same throughout the disc due to its small contribution to the complete disc stiffness compared to the fibers and its value was taken from the literature^63,64^. The intrinsic viscoelasticity of the ECM was also considered regional independent. The set of ECM material constants is:

15 $$ \left\{ {E,\;E_{v} ,I_{1}^{\max } ,I_{1v}^{\max } ,d,m} \right\} = \left\{ {{\text{1 MPa}}, \, 0.{\text{167 MPa}},{\text{ 3 MPa}},{ 1}.{\text{5 MPa}}, \, 0.0{\text{2 MPa}}^{{ - {1}}} \;{\text{s}}^{{ - {1}}} , \, 0.00{1}} \right\} $$

The effect of CFs is considered to be mainly governed by their content and orientation. The CFs intrinsic mechanical parameters would not change for the different disc regions:

16 $$ \left\{ {A_{1} ,A_{2} } \right\} = \left\{ {{\text{27 MPa}},{ 1}00{\text{ MPa}}} \right\} $$
